# Coordinating smoking cessation treatment with menstrual cycle phase to improve quit outcomes (MC-NRT): study protocol for a randomized controlled trial

**DOI:** 10.1186/s13063-023-07196-1

**Published:** 2023-04-01

**Authors:** Laurie Zawertailo, Tina Kabir, Sabrina Voci, Elise Tanzini, Sophia Attwells, Liliana Malat, Scott Veldhuizen, Nadia Minian, Rosa Dragonetti, Osnat C. Melamed, Elad Mei-Dan, Peter Selby

**Affiliations:** 1 INTREPID Lab (formerly Nicotine Dependence Service), Centre for Addiction and Mental Health, 1025 Queen St. W, Toronto, Ontario M6J 1H4 Canada; 2 Department of Pharmacology and Toxicology, University of Toronto, 1 King’s College Circle, Toronto, Ontario M5S 1A8 Canada; 3 Campbell Family Mental Health Research Institute, Centre for Addiction and Mental Health, 100 Stokes St., Toronto, Ontario M6J 1H4 Canada; 4 Department of Psychiatry, University of Toronto, 250 College St., Toronto, Ontario M5T 1R8 Canada; 5 Department of Family and Community Medicine, University of Toronto, 500 University Ave., Toronto, Ontario M5G 1V7 Canada; 6 North York General Hospital, 4001 Leslie St., Toronto, Ontario M2K 1E1 Canada; 7 Dalla Lana School of Public Health, 155 College St., Toronto, Ontario M5T 3M7 Canada

**Keywords:** Smoking cessation, Menstrual cycle, Ovarian hormones, Estrogen, Progesterone, Smoking cessation treatment, Nicotine replacement therapy, Cancer prevention, Nicotine dependence

## Abstract

**Background:**

Women experience greater difficulty achieving smoking abstinence compared to men. Recent evidence suggests that hormonal fluctuations during different phases of the menstrual cycle can contribute to lower smoking abstinence rates following a quit attempt among women. However, these findings are limited by small sample sizes and variability among targeted smoking quit dates. This clinical trial aims to clarify whether targeting the quit date to the follicular or luteal phase of the menstrual cycle can improve smoking abstinence.

**Methods:**

Participants will enroll in an online smoking cessation program providing nicotine replacement therapy (NRT) and behavioral support. We will randomize 1200 eligible individuals to set a target quit date: (1) during the mid-luteal phase, (2) during the mid-follicular phase, or (3) 15–30 days after enrollment with no regard to the menstrual cycle phase (usual practice). Participants will receive a 6-week supply of combination NRT consisting of a nicotine patch plus their choice of nicotine gum or lozenge. Participants will be instructed to start using NRT on their target quit date. Optional behavioral support will consist of a free downloadable app and brief videos focusing on building a quit plan, coping with cravings, and relapse prevention, delivered via e-mail. Smoking status will be assessed via dried blood spot analysis of cotinine concentration at 7 days, 6 weeks, and 6 months post-target quit date.

**Discussion:**

We aim to overcome the limitations of previous studies by recruiting a large sample of participants and assigning target quit dates to the middle of both the follicular and luteal phases. The results of the trial can further elucidate the effects of the menstrual cycle on smoking cessation outcomes and whether it is beneficial to combine menstrual cycle phase timing strategies with accessible and low-cost NRT.

**Trial registration:**

ClinicalTrials.gov NCT05515354. Registered on August 23, 2022.

**Supplementary Information:**

The online version contains supplementary material available at 10.1186/s13063-023-07196-1.

## Administrative information

**Table:** 

Title {1}	Coordinating smoking cessation treatment with menstrual cycle phase to improve quit outcomes: a randomized controlled trial
Trial registration {2a and 2b}	Registered at ClinicalTrials.gov, NCT05515354, Date of registration: 08.23.2022
Protocol version {3}	Version date: October 4, 2022. Version number: 1.2.
Funding {4}	This study is funded through a grant from the Canadian Cancer Society Research Institute (CCS), Grant # 707321-1. St. Clair Ave W, Suite 300, Toronto, Ontario, M4V 2Y7, Canada 416-961-7223
Author details {5a}	^1^INTREPID Lab (formerly Nicotine Dependence Service), Centre for Addiction and Mental Health, 1025 Queen St. W, Toronto, Ontario, M6J 1H4, Canada ^2^Department of Pharmacology and Toxicology, University of Toronto, 1 King’s College Circle, Toronto, Ontario, M5S 1A8, Canada ^3^Campbell Family Mental Health Research Institute, Centre for Addiction and Mental Health, 100 Stokes St., Toronto, Ontario, M6J 1H4, Canada ^4^Department of Psychiatry, University of Toronto, 250 College St., Toronto, Ontario, M5T 1R8, Canada ^5^Department of Family and Community Medicine, University of Toronto, 500 University Ave., Toronto, Ontario, M5G 1V7, Canada ^6^Dalla Lana School of Public Health, 155 College St., Toronto, Ontario, M5T 3M7, Canada ^7^North York General Hospital, 4001 Leslie St., Toronto, Ontario, M2K 1E1, Canada
Name and contact information for the trial sponsor {5b}	Centre for Addiction and Mental Health (CAMH), Addictions Program is the sponsor of the study: 100 Stokes St., Toronto, Ontario M6J 1H4, Canada 416-535-8501
Role of sponsor {5c}	The sponsor has the ultimate authority on the study design and conduct. The research team must comply with standards of operation (SOPs) established by the sponsor. The sponsor makes decisions about trial discontinuation, conducts audits and inspections of the site and all study-related documents, has exclusive full access to the final trial dataset, and owns all data and results, excluding personal health identifiers (PHI). The sponsor controls the release of clinical trial information and dissemination of study results. The funder (CCS) has no role in the design of the study or the collection, analysis, interpretation of the data and production of this manuscript.

## Introduction

### Background and rationale {6a}

Tobacco use is a risk factor for at least 20 types of cancer [1] and remains the leading preventable cause of cancer in Canada [2]. In 2015, an estimated 15% of all cancer diagnoses in Canadian women were attributable to active tobacco smoking, including 69% of lung cancer cases and 26% of cervical cancer cases [2]. Thus, smoking cessation is an important cancer prevention strategy for the close to 2 million Canadian women who currently smoke [3]. Although quitting smoking at any age is beneficial, women who quit smoking before age 45 avoid approximately 90% of lung cancers and overall mortality seen among continuing smokers [4, 5].

Although 45% of all smokers in Canada report making at least one quit attempt in the past year, 88% of these quit attempts are unsuccessful [6]. Evidence from both controlled trials and real-world clinical settings indicate that women have even greater difficulty achieving abstinence following a quit attempt than men [7, 8]. Our research group recently conducted a secondary analysis of data from 27,601 participants enrolled in our Smoking Treatment for Ontario Patients (STOP) program that confirmed significantly lower quit success in women undergoing smoking cessation treatment compared to men (24% vs. 27%) [9]. On a population level, this percentage difference translates into thousands of Canadian women continuing to accrue the risk of developing cancer and other smoking-related diseases. Thus, strategies are needed to increase the effectiveness of smoking cessation treatment for women to decrease the occurrence of preventable cancers and premature mortality.

In our STOP program, which provides up to 26 weeks of nicotine replacement therapy (NRT) personalized to individual needs, we found that associations between quit success and sex were not modified by the formulation, dose, and duration of NRT received. Additionally, the difference was independent of other confounding variables such as health and sociodemographic factors [9]. Thus, although women using NRT had lower overall quit success than men, variations in the type or duration of NRT received neither strengthened nor diminished this association between gender and quit outcome. This finding suggests that other factors, such as hormonal changes, need to be prioritized to help women using NRT achieve similar levels of quit success as men.

Individuals with a naturally occurring menstrual cycle experience changes in ovarian hormone levels, estrogen, and progesterone, across menstrual cycle phases. There is increasing evidence for the contribution of fluctuating ovarian hormones to the greater difficulty that women experience when trying to quit smoking [10]. As such, targeting quit date to the menstrual cycle phase could be a simple and effective strategy to improve smoking quit outcomes among premenopausal individuals.

In this study, we will test targeting initiation of a quit attempt assisted with NRT to specific phases of the menstrual cycle with a sample of premenopausal individuals. We hypothesize that starting a quit attempt during the follicular phase will result in increased quit success compared to starting during the luteal phase or the usual practice of not targeting quit start date to a menstrual cycle phase.

#### Clinical data supporting study interventions

A systematic review of menstrual cycle effects on nicotine withdrawal and craving following smoking cessation found that significantly greater withdrawal, and a trend for greater craving, was reported by women during the luteal phase compared to the follicular phase of their menstrual cycle [10]. In a pilot study of 35 female smokers who made a quit attempt with the aid of nicotine patch and counseling, those randomized to quit during their follicular phase (high estrogen) vs. luteal phase (high progesterone) had higher rates of abstinence 2 weeks after their quit date (32% vs. 19%), although the difference was non-significant [11]. Similarly, a second study [12] of 102 women receiving 8 weeks of NRT plus behavioral support found significantly higher quit rates on day 3 of treatment (81% vs. 48%) and 1 week after the end of treatment (69% vs. 29%) among women who set their quit date during the follicular phase versus the luteal phase. However, another study found rising progesterone levels (highest during the luteal phase) were associated with a 37% increase in odds of abstinence for the group that received nicotine patch (*n* = 56) [13]. These findings suggest that ovarian hormones and the menstrual cycle phase have an impact on NRT-aided quit outcomes. However, evidence for the influence of the menstrual cycle phase on smoking cessation outcomes have been limited by small samples and a lack of clarity surrounding ideal quit date timing. Therefore, we plan to conduct an adequately powered, rigorously designed study to clarify whether targeting quit date to the follicular or luteal phase of the menstrual cycle improves quit success*.*

#### Risks and benefits of study interventions

Regardless of which group a participant is assigned to, the study intervention will increase participants’ chances of successfully quitting smoking. Quitting smoking is the single most effective thing one can do to decrease their risk of preventable disease and death. NRT, such as nicotine patches, gum, and lozenge, is available over the counter in Canada because the risks of using NRT are minimal. The most common side effect of the nicotine patch is itchiness and redness at the site of the patch application. The most common side effect of nicotine gum or lozenge is upset stomach from swallowing the nicotine rather than letting it absorb through the buccal mucosa.

### Objectives {7}

The *primary objective* of this study is to determine whether targeting the quit date to either the luteal or follicular menstrual cycle phase improves short-term quit outcomes (7 days post-target quit date) compared to a self-selected target quit date without regard to the menstrual cycle phase (usual practice) for premenopausal persons provided with NRT and behavioral support to quit smoking.

The *secondary objectives* are (1) to determine whether there is a difference in short-term quit outcomes (7 days post-target quit date) between the groups targeting quit date to the follicular versus luteal phase of the menstrual cycle and (2) to identify any group differences in quit outcomes at the end of treatment (6 weeks) and 6-month follow-up.

### Trial design {8}

The study design is an open-label, parallel-group, superiority randomized controlled trial. Participants will be randomized into three groups in a 1:1:1 allocation ratio: (1) quit smoking during the mid-luteal phase of their menstrual cycle (6–8 days pre-onset of menses), (2) quit smoking during the mid-follicular phase of their menstrual cycle (6–8 days post-onset of menses), or (3) quit smoking 15–30 days post-enrollment with no regard to the menstrual cycle phase (usual practice). All participants will receive 6 weeks of NRT to aid in their quit attempt. The expected duration of the clinical trial is 48 months; we expect participant recruitment and data collection to occur over 27 months. Participants are not required to make any in-person visits; therefore, any potential COVID-19 restrictions are not expected to cause any significant disruptions to our timeline.

## Methods: participants, interventions, and outcomes

### Study setting {9}

The study will be conducted at Nicotine Dependence Services (NDS) at the Centre for Addiction and Mental Health (CAMH) in Toronto, Canada. Notably, this study is an Internet-based trial, and all interactions with study participants, including collection of informed consent, delivery of study instructions, baseline, and follow-up assessments, will be conducted virtually using Research Electronic Data Capture (REDCap) [14], a partner platform operated by CAMH, as well as via email and phone/text communication.

### Eligibility criteria {10}

#### Inclusion criteria

The study will recruit 1200 treatment-seeking naturally cycling individuals within Ontario, with a regular menstrual cycle (defined as cycle length ranging from 21 to 35 days over the past 6 months) aged 18 to 40 years. Eligible individuals must smoke at least 5 cigarettes per day over the past 6 months, be willing to quit smoking using NRT (nicotine patches and nicotine gum or lozenge) within 30 days and to make a quit attempt on their assigned target quit date, be willing to comply with study procedures (including providing dried blood spots), and provide a valid e-mail address to receive essential study communication and invitations to complete follow-up questionnaires.

#### Exclusion criteria

Individuals will be excluded from the study if they meet at least one of the following criteria: currently using progesterone, estrogen, testosterone, or fertility treatment; currently using NRT or other smoking cessation medications (e.g., varenicline, bupropion, cytisine); have used hormonal contraceptives in the past 6 months (e.g., pill, patch, hormonal intrauterine device [IUD], ring); are pregnant or trying to become pregnant within the next 3 months; have known hypersensitivity or allergies to any of the components of the nicotine patch; have used cannabis daily or almost daily in the past 30 days; have used tobacco or nicotine products other than cigarettes (e.g., smokeless tobacco, heat-not-burn products, e-cigarettes) daily or almost daily in the past 30 days; were diagnosed with polycystic ovary syndrome; have an unstable psychiatric condition (including substance use disorder) which would compromise study compliance; suffer from life-threatening arrhythmias or severe/worsening angina pectoris; experienced myocardial infarction or cerebral vascular accident in the past 2 weeks; or were diagnosed with a terminal illness.

### Who will take informed consent? {26a}

As this study is an Internet-based trial, participants will document their informed consent independently using a modified REDCap e-Consent Framework [15]. When potential participants indicate their interest in the study, they will be directed to read the consent and explore the FAQ website page related to the informed consent and the study. Potential participants will also be provided with study contact information (email address and phone number) and encouraged to reach out to discuss the study consent and ask any questions. They will be advised not to provide their consent until all their questions have been answered to their satisfaction.

### Additional consent provisions for collection and use of participant data and biological specimens {26b}

Consent on the use of data in ancillary studies is provided with the informed consent for the study.

### Interventions

#### Explanation for the choice of comparators {6b}

The usual practice was selected as the comparator condition in order to detect whether the anticipated benefit of targeting quit date to a particular menstrual cycle phase produces better quit outcomes than the current practice. Although targeting quit date to the follicular phase may lead to greater quit success than targeting quit date to the luteal phase (or vice versa), a benefit over usual practice is needed to support a change in the current treatment approach.

#### Intervention description {11a}

Participants will enroll in an online smoking cessation program providing NRT and behavioral support. Participants will receive a 6-week supply of NRT consisting of a nicotine patch plus their choice of nicotine gum or lozenge, which they will be instructed to start on their target quit date. Participants will be randomized to set a target quit date: (1) during the mid-luteal phase of their menstrual cycle (6–8 days pre-onset of menses), (2) during the mid-follicular phase of their menstrual cycle (6–8 days post-onset of menses), or (3) 15–30 days post-enrollment with no regard to the menstrual cycle phase (usual practice). We are allowing 14 days for the delivery of the NRT package. Hence, the earliest target quit date that a participant can have is at 15 days post-enrollment. Depending on the group they are randomized to, some participants may need to wait until their next cycle to start their quit attempt. Target quit date windows for all participants will be calculated in accordance with their menstrual cycle information (start date of last period and usual cycle length) and their enrollment date. Behavioral support will consist of a free downloadable app (My Change Plan) and brief videos delivered via e-mail. These videos have been produced by the CAMH NDS and will focus on health behavior change strategies, such as building a quit plan, coping with cravings, and relapse prevention [16].

The research team will mail each participant a study kit consisting of a 6-week supply of NRT (patches plus gum or lozenge) and dried blood spot collection kits. Participants who smoke 10 or more cigarettes per day will receive 4 weeks of 21 mg and 1 week each of 14 mg and 7 mg nicotine patches, while those smoking 5–9 cigarettes per day will receive 4 weeks of 14 mg and 2 weeks of 7 mg nicotine patches. Nicotine patches will be pre-organized into weekly packages, requiring the participants to reduce the dosage from 21 mg or 14 mg to 7 mg throughout the 6-week treatment period. All groups will be instructed to collect a dried blood spot and start using their NRT on their target quit date, as well as attempt to be completely abstinent from smoking starting from their target quit date onwards. Dried blood spots will be used to measure blood cotinine levels to confirm participants’ smoking status. Participants will be instructed to apply a new patch every day and use 2 mg nicotine gum or lozenge as needed to curb any urges to smoke. The research team will provide each participant with 2 packs of nicotine gum in which the maximum dosage is 30 pieces a day. These combinations and dosages of NRT have been found most effective for smoking cessation [17].

**Fig. 1:**
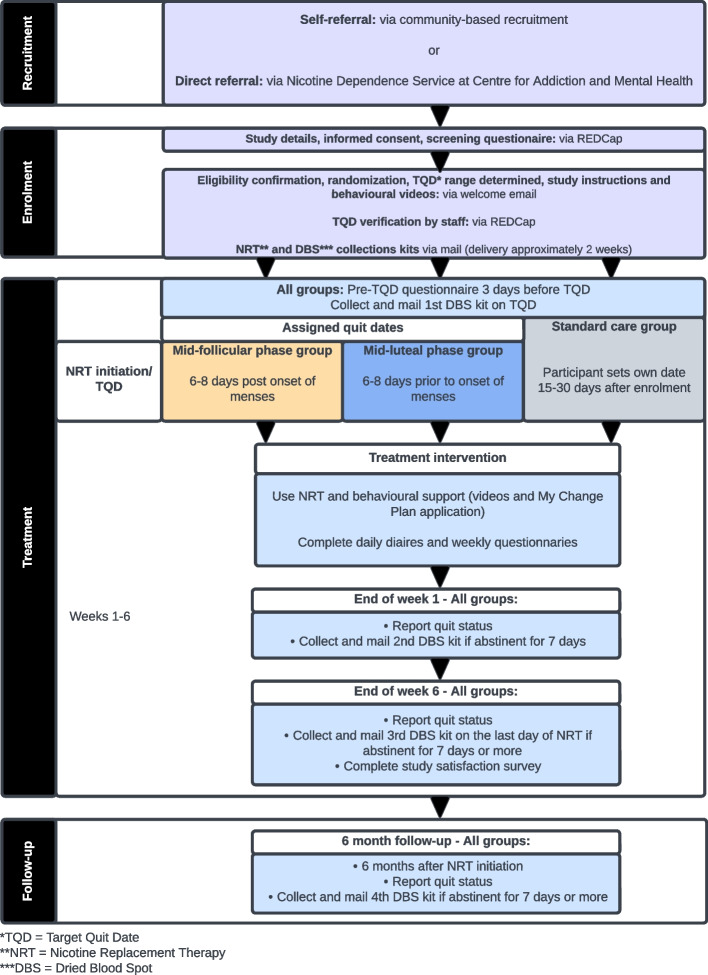
Flow diagram for study events

**Fig. 2:**
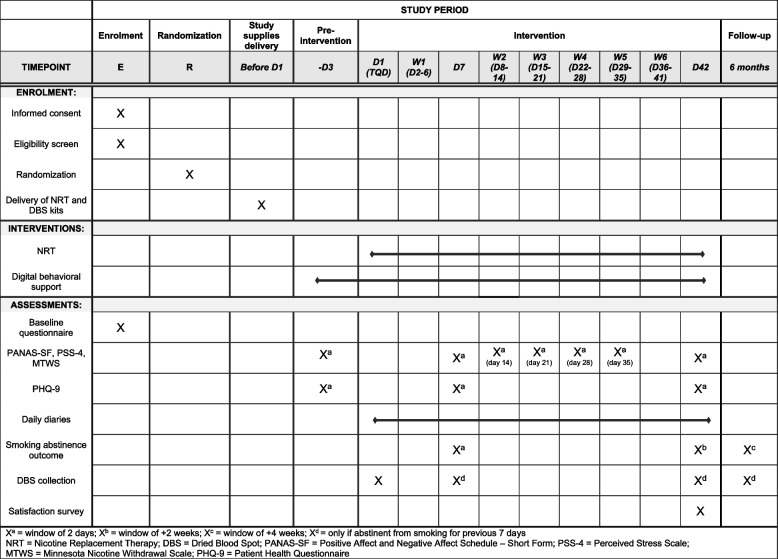
Schedule of study events for each participant (SPIRIT figure)

Participants will also have access to the behavioral support videos and handouts from the time they receive their welcome e-mail until the end of the study. Participants will be asked to complete daily diaries and weekly questionnaires to document tobacco use, NRT use, nicotine craving and withdrawal symptoms, mood, adverse events, and menstrual cycle symptoms via online REDCap surveys. Additional questionnaires will include Patient Health Questionnaire [18] (PHQ-9; weeks 1 and 6), Positive Affect and Negative Affect Schedule – Short Form [19] (PANAS-SF; weeks 1–6), Minnesota Nicotine Withdrawal Scale [20] (MNWS; weeks 1–6), and Perceived Stress Scale 4 [21] (PSS-4; weeks 1–6). Smoking status will be assessed at the end of weeks 1 and 6; those who self-report continuous abstinence from smoking for at least 7 previous days will be asked to collect dried blood spots and mail them within 3 days of collection (for biochemical verification of abstinence). At the end of treatment (week 6), participants will also be asked to complete a study satisfaction survey. Refer to Fig. 1 for a brief overview of the study and to Fig. 2 for a detailed schedule of events.

#### Criteria for discontinuing or modifying allocated interventions {11b}

Participants are free to discontinue the study intervention at any time. The study team will be able to identify the discontinuation when a participant does not complete follow-up surveys.

NRT is an over-the-counter product with a demonstrated safety profile. Therefore, we anticipate that the risk to participant safety will be minimal. However, participants may experience adverse events that can affect their decision to complete the intervention. The most common side effect of the nicotine patch is rash at the site of the patch application. This reaction can be quite pronounced in some people and may preclude their continued use of the patch. Other side effects include sleep disturbance and signs of nicotine toxicity. In case of severe sleep disturbance, it is recommended that participants take the patch off for 1–2 h before bed. If a participant is experiencing signs of nicotine toxicity, they are instructed to take the patch off for a few hours, switch to patches with lower nicotine content, or use nicotine gum/lozenge less frequently. Regardless, participants will be told to stop using the NRT if they experience adverse events that are not acceptable to them. The participants can still use nicotine gum or lozenge to aid in their quit attempt as well as the behavioral resources. We will ask participants to report their daily NRT use or non-use. We do not anticipate any systematic differences in the discontinuation of the intervention among the 3 groups since the intervention is the same for all groups.

#### Strategies to improve adherence to interventions {11c}

Daily diaries and weekly questionnaires will be programmed for automatic delivery in REDCap, based on the target quit date of the participant. Participants will also be notified about their target quit date and receive reminders to complete their baseline assessments, daily diaries, and weekly questionnaires. Reminders and notifications will be sent via email and/or SMS texts. If a participant does not provide information pertaining to the primary outcome of the study (quit status on day 7), the study staff will attempt to follow up with them.

As an incentive to complete and mail back dried blood spot collections, we will compensate participants with a $5 e-gift card for each dried blot spot collection received. Thus, all participants are eligible to receive at least one $5 e-gift card (baseline assessment) and can receive up to a total of $20 in e-gift cards throughout the study (up to 4 blood spots can be collected depending on whether participants achieve abstinence from smoking).

#### Relevant concomitant care permitted or prohibited during the trial {11d}

The screening questionnaire asks participants about the use of smoking cessation medications (e.g., varenicline and bupropion) that exclude them from participating in the study. Participants are also invited to disclose any medication use at baseline and week 6.

#### Provisions for post-trial care {30}

The interventions are designed to inflict minimal to no harm. If a participant is not successful in quitting within the study period and would like to explore other options for assistance with quitting, they may be directed to other CAMH programs and services, such as the Nicotine Dependence Clinic and STOP on the Net program.

### Outcomes {12}

#### Primary outcome

We are primarily interested in the between-group comparison of proportions of biochemically confirmed abstinence, measured through dried blood spots, at 7 days post-target quit date. The trial will be considered successful if the proportion of abstinent participants in either of the groups with a target quit date based on the menstrual cycle phase (follicular or luteal) is significantly higher than in the usual practice group.

#### Secondary outcomes

The secondary outcomes of the study are potential differences in proportions of biochemically confirmed abstinence (1) between the two intervention arms targeting quit date to menstrual cycle phase at 7 days and (2) between the two intervention arms targeting quit date to menstrual cycle phase and usual practice at 6 weeks and 6 months post-target quit date.

### Participant timeline {13}

#### Informed consent, screening, and baseline assessment

Following direct or self-referral to the study, potential participants will be provided with detailed study information and will be asked to provide informed e-consent. Participants will then be asked to complete a screening questionnaire to determine eligibility, and those who are eligible will be automatically directed to complete a baseline assessment to collect additional background information (e.g., sociodemographic, tobacco use history) and a contact information questionnaire to collect the participant’s mailing address, email, and phone number.

#### Randomization and study instructions

Enrolled participants will be randomized to one of three groups (*n* ~ 400 per group). A welcome email will inform participants of their target quit date range and ask them to inform study staff of their planned quit date (within the range) and begin their NRT on this date. The email will also provide instructions on how to use the NRT and dried blood spot kits and will contain Purolator shipment tracking information to track the delivery of the study kit. The email will also provide information about the online daily diaries, weekly questionnaires, and the 6-month follow-up questionnaire. Lastly, the email will include a link to the behavioral support resources and handouts.

#### Pre-target quit date assessment

Three days before their target quit date, participants will be sent a link to complete questionnaires to assess their recent mood (PHQ-9 [18] and PANAS-SF [19]), nicotine withdrawal symptoms (MNWS [20]), and level of perceived stress (PSS-4 [21]).

#### Treatment intervention (6 weeks)

Participants will undergo 6 weeks of NRT treatment with access to additional behavioral support via videos and My Change Plan smartphone app. The timing of the start of NRT to a particular menstrual cycle phase is part of the intervention.

#### Follow-up period (6 months)

At 6 months post-target quit date, participants will complete a follow-up questionnaire regarding current smoking status and smoking behavior since study participation to capture the incidence of lapse and relapse. If they self-report abstinence for at least 7 days, they will be mailed a fourth dried blood spot kit, asked to collect the dried blood spot, and mail it within 3 days of collection. For a schematic presentation of the participant timeline, refer to Fig. 2.

### Sample size {14}

We estimate 1200 participants (1:1:1 allocation ratio) to be a realistic sample size, allowing us to achieve sufficient statistical power for the primary outcome analysis. To evaluate power for the primary objective, we ran simulations at various sample sizes by randomly generating data using distributions and coefficients drawn from existing data from the STOP Program. To confirm the final sample, we ran a set of 10,000 simulations. A total analysis sample of 1200 participants allocated in a 1:1:1 ratio using simple randomization will provide 80% power to detect a statistically significant difference for our primary outcome. We anticipate that the proportions quitting successfully on day 7 post-TQD will be 25% in the usual practice group, 34% in one intervention arm, and 25% in the other intervention arm. The effect sizes were calculated without accounting for attrition in the final sample size.

### Recruitment {15}

The Nicotine Dependence Clinic at CAMH offers in-person and online smoking cessation programs and will be one source of referral to the study. Our STOP on the Net online smoking cessation program enrolls 1500–2000 participants per month, on average, which includes 300–400 self-identified women aged 18–40, illustrating the feasibility of recruiting 1200 eligible women over 2 years to participate in this study. The trial will be posted on the public CAMH website including the “CAMH Find a Study online” where individuals can explore studies that they may be interested in participating in. In addition, participants can learn about the study through online postings (i.e., Google Ads, Facebook, Twitter, Instagram) and word-of-mouth. In total, we expect to enroll at least 70 eligible participants per month and have the capacity to enroll as many as 200 per month.

### Assignment of interventions: allocation

#### Sequence generation {16a}

Given the large sample size, the need to assign groups at the time of enrollment, and the expense and complexity of implementing more complex strategies in an online platform, we will allocate participants to treatment groups using simple randomization, implemented through the randomization module in REDCap. Randomization parameters were defined, and an allocation table was developed by the study team outside of REDCap.

#### Concealment mechanism {16b}

Randomization of each participant will occur shortly after their enrollment. Participants will not be directly informed of their group assignment; however, they will be provided with a date range for their target quit date to prepare for their quit attempt and the start of the intervention. The allocation sequence will be uploaded to REDCap and will be protected by restricting user rights so it will only be accessible to the study statistician and data manager, who are not involved in recruitment. Therefore, the sequence will be concealed from both participants and unauthorized study staff.

#### Implementation {16c}

The allocation sequence will be generated using a computer application for random number generation and uploaded to REDCap. Following enrollment of eligible participants (upon completion of the informed consent form and screening questionnaire), the research staff will allocate each participant to a group using the REDCap randomization module.

### Assignment of interventions: blinding

#### Who will be blinded {17a}

This study is open-label. However, the data analysis and outcome adjudication will be blinded.

#### Procedure for unblinding if needed {17b}

Once the analysis of the primary and secondary outcomes is complete, the identity of the three groups will be revealed.

### Data collection and management

#### Plans for assessment and collection of outcomes {18a}

Quit status at day 7, week 6, and 6 months post-target quit date will be assessed using REDCap questionnaires designed by the research team (see Additional file 1). These repeated assessments at different time points will allow us to track the participants’ quit status longitudinally and assess both short- and long-term outcomes. If a participant reports abstinence from smoking, their abstinence will be confirmed biochemically using dried blood spot analysis. Collecting dried blood spots is minimally invasive, cost-effective, and ideal for home sampling when conducting population-based research [22]. Filter paper blood collection demonstrates the same level of precision and reproducibility as standard methods of collecting blood [23], and the results are precise for cotinine [24], progesterone, and estradiol [25]. Additionally, the participants will be directed to complete daily diaries designed by the research team in REDCap (see Additional file 2) to provide data about their day-to-day smoking status and NRT use. The participants will also complete baseline and weekly standardized assessments of mood (PHQ-9 [18] and PANAS-SF [19]), nicotine withdrawal symptoms (MNWS [20]), and perceived stress (PSS-4 [21]). An analysis plan for daily diaries and standardized assessments is currently being developed.

#### Plans to promote participant retention and complete follow-up {18b}

Participant retention will be promoted through regular email and SMS reminders to complete study assessments. Completion of dried blood spot assessments will be encouraged through compensation in the form of $5 e-gift cards for each submitted dried blood spot. We expect to see some missing data, which will be accounted for in the statistical analysis. If a participant demonstrates significant non-adherence (defined as failure to complete week 1 and 6 questionnaires and provide dried blood spots at these time points, if required), they will be withdrawn from the study, and their data will not be retained. If a participant withdraws from the study voluntarily, a reasonable effort will be made to determine the reason for withdrawal; their data will not be retained.

#### Data management {19}

The PI and study personnel will be responsible for managing data collection. As this is an Internet-based trial, the participants will complete assessments individually, with their data entered directly into REDCap. Administered surveys will predominantly collect quantitative data. Dried blood spot samples will be analyzed at a biochemical laboratory affiliated with the University of Toronto.

Data for this study will be managed using REDCap electronic case report forms. This system is maintained on central CAMH servers, with data backed up daily, and is supported by the Research Informatics department. Access to study data will be limited to the PI and primary research team members. In order to ensure that participant information is only accessible to approved members of the study team, REDCap permissions to access the project data will only be provided to the users who need to access the data for study conduct or monitoring purposes. A data dictionary (and any other necessary supporting documentation) will be created and saved along with the datasets, so that others can understand the variables and data structure.

Once data collection is complete, the data will be backed up on a CAMH server to a folder with limited access, and the necessary supporting documentation will be saved along with the files. Only the PI, and other members of the study team who require direct access to the data for data management and/or analysis, will be given permission to access this folder.

The PI will retain all study data for a period of at least 10 years as per CAMH policy for unregulated studies.

#### Confidentiality {27}

Personal identifying information will only be collected once at the time of enrollment for the purposes of mailing study intervention supplies to participants and communicating survey invitations via email or text, depending on participant preference. Each randomized participant will be assigned a unique identifier that will be used to link all the questionnaires for that participant. The personal identifying information will be kept separate from the participant data. Materials used to mail back dried blood spot collections for testing, and test results, will be labeled with a participant identification number only and not with any personal identifying information. For daily diary and weekly questionnaires, participants will be given the option to receive SMS text messages via Twilio. Twilio is a widely used third-party SMS provider integrated with the REDCap platform. Phone number and mailing address are kept separately from other participant information and are accessed from a de-identified record by Twilio and study team to send SMS and mail out the study packages, respectively.

After data collection is complete, the data will be exported from REDCap in csv file format, to permit data analysis, sharing, and storage on CAMH’s secure Y-drive. A single master .csv file with personal identifying information will be saved separately from a single master .csv file with all de-identified study data. Master files will not be used for analysis to minimize the risk of data being improperly or erroneously overwritten or modified. A copy of the master de-identified file will be used for all analyses, and syntax will be used to select and modify cases and variables without saving, to avoid creating several versions of the same dataset. The copy of the master dataset will be saved in the appropriate file format for the program being used for analysis and data visualization (e.g., Stata); multiple copies may be saved if multiple programs are used.

#### Plans for collection, laboratory evaluation, and storage of biological specimens for genetic or molecular analysis in this trial/future use {33}

Dried blood spot will be collected on the first and last days of NRT treatment and on day 7 of NRT treatment. Dried blood spots will determine the progesterone and estrogen levels to confirm the phase of the menstrual cycle and determine cotinine concentration levels to confirm smoking status. Participants will perform the collection procedure independently. Drops of whole blood are collected on filter paper from a finger prick with a single-use lancet following sterilization with isopropyl alcohol. Blood drops are left to dry for at least 4 h or overnight, and the filter paper matrix stabilizes the analytes in the dried blood spot at room temperature [22]. Dried blood spots provided by the participants will be stored at − 80 °C in the freezer at a designated CAMH facility prior to biochemical analysis at a University of Toronto-affiliated laboratory and will be destroyed after they are analyzed.

## Statistical methods

### Statistical methods for primary and secondary outcomes {20a}

#### Primary analysis

To evaluate the primary outcome, we will compare the proportions of biochemically abstinent participants between the groups. Consistent with current practice, and because we expect an advantage over usual practice for only one menstrual cycle phase group, we will compare each intervention arm to usual practice without adjustment for multiplicity. A *p*-value < 0.05 will be considered statistically significant.

We will use logistic regression to test the group differences. As adjustment for prognostically important baseline covariates increases power and decreases bias, we will adjust for age, income, and the heaviness of smoking index (HSI), all of which are known to be meaningfully predictive of successful smoking cessation. We will fit two separate models [26], one for each of our comparisons (i.e., follicular phase group vs. usual practice group and luteal phase group vs. usual practice group).

#### Secondary analyses

To test for a difference between the two intervention arms, we will fit a third model comparing day 7 post-target quit date outcomes in the follicular phase and luteal phase group. To test the group differences at later time points, we will use mixed-effects logistic regression with a random person-level intercept. We will incorporate all follow-ups and all groups into a single model. If within-group change can be captured by a linear slope and an interaction, we will fit a model including group, time, group × time, age, income, and HSI as fixed effects. Otherwise, we will dummy-code time points and interact these with group indicators. In either case, we will derive and test group differences at specific time points using post-estimation procedures.

### Interim analyses {21b}

There are no interim analyses and stopping guidelines planned for this study. PI and sponsor can make the final decision to terminate the study.

### Methods for additional analyses (e.g., subgroup analyses) {20b}

Secondary analyses and subgroup analyses are currently being developed. We are planning to do subgroup analyses of standardized assessment data (e.g., mood and craving), daily diaries, and NRT use.

### Methods in analysis to handle protocol non-adherence and any statistical methods to handle missing data {20c}

We anticipate minimal missing data (< 5%) for the primary outcome (day 7 smoking status) due to our daily contact with participants during the treatment phase. We will evaluate the effects of missingness by calculating differences across plausible and extreme missing-data scenarios. If our primary test is significant, we will also calculate the size of the interaction between the group and missingness that would be required to make this difference non-significant. If missing data causes the exclusion of 10% or more of the baseline sample, we will conduct an analysis using multiple imputation. In our imputation models, we will include the variables from our primary analysis, biochemically verified quit status, data on craving, and NRT use at all available time points.

### Plans to give access to the full protocol, participant-level data, and statistical code {31c}

Anonymized participant data may be shared for use in other studies. There are no intentions to grant public access to protocol and statistical code.

### Oversight and monitoring

#### Composition of the coordinating center and trial steering committee {5d}

The day-to-day management group will consist of the PI, a doctoral student, a research coordinator, an assistant manager, and seasonal research placement students. The group will meet weekly. This study does not have a Trial Steering Committee.

#### Composition of the data monitoring committee, its role, and reporting structure {21a}

A Data Monitoring Committee (DMC) is not required, as there are no interim analyses, no planned procedures for early stopping, and the treatment used (NRT) is available over the counter.

#### Adverse event reporting and harms {22}

Adverse events will be collected daily and weekly via REDCap surveys and reviewed by the study investigators for any serious or unexpected adverse events. Applicable serious unexpected adverse events (SAEs) will be recorded and reported to REB in accordance with REB requirements and timelines.

#### Frequency and plans for auditing trial conduct {23}

The PI and site will permit study-related audits, and inspections by the REB, CAMH, sponsor, and applicable granting agencies or regulatory bodies, including access to all study-related documents (e.g., source documents, regulatory documents, data collection instruments, study data). The PI will ensure the capability for audits/inspections of applicable study-related facilities (e.g., research pharmacy, clinical laboratory, imaging facility).

#### Plans for communicating important protocol amendments to relevant parties (e.g., trial participants, ethical committees) {25}

No deviations from or changes to the protocol will be implemented without prior agreement from the sponsor as required, and approval from the REB, unless to eliminate an immediate hazard to a participant.

#### Dissemination plans {31a}

The PI will hold primary responsibility for the publication of the results of this clinical trial. Publications will be prepared in compliance with CAMH Policy AR 1.17 (Research Publications).

## Discussion

The majority of quit attempts are unsuccessful [6], with women demonstrating lower quit success than men [9]. This study aims to close this gap by investigating a new intervention to improve quit outcomes in individuals with menstrual cycles. We propose an intervention of coordinating the start date of NRT either to the mid-follicular or mid-luteal phase of the menstrual cycle. Although there is a body of research on smoking cessation relative to the menstrual cycle, previous studies report equivocal findings and are limited by small samples and a lack of clarity regarding ideal quit date timing [11–13]. This trial will overcome these limitations by recruiting a large sample of participants and limiting the target quit date to the middle of both the follicular and luteal phases. Through this online trial with minimally invasive interventions, participants will have free access to long- and short-acting NRT along with behavioral support, proven to be one of the most effective combination treatment strategies for smoking cessation (with the two interventions demonstrating higher efficacy when used together, rather than alone) [27, 28]. Therefore, participants will have a higher chance of quitting relative to unaided attempts, regardless of group allocation. The timing interventions will be compared to usual practice. The results of the trial can inform the recommendations to further explore the effects of the menstrual cycle on smoking cessation outcomes. The findings could also provide evidence for the implementation of menstrual cycle phase timing strategies in smoking cessation programs.

The challenge that the trial team has encountered so far is a delay in the trial timeline, resulting from an extended time period to get REB approval and prepare for participant recruitment. Some potential future limitations of this study may include loss to follow-up and missingness of data due to the online nature of the study. Loss to follow-up can be prevented by email and SMS reminders, as well as gift card incentives to complete dried blood spot collections. Missingness of the data will be accounted for in the statistical analysis.

Overall, coordinating the start of NRT to the menstrual cycle phase can be an easy, accessible, low-risk, and low-cost method of improving the quit success rate in a large proportion of smokers. This randomized clinical trial can inform further preventative measures to improve smoking outcomes in premenopausal individuals, therefore having a positive impact on the incidence of smoking-related disease.

## Trial status

The original version of the protocol (version 1.1 dated May 6, 2022) was approved by CAMH REB on June 9, 2022. The ethical approval document is provided in Additional file 3. Current version 1.2 (dated October 4, 2022) was approved by CAMH REB on November 8, 2022. The trial was registered on ClinicalTrials.gov on August 23, 2022. Recruitment commenced on November 30, 2022, and is expected to be completed in 2024.

## Supplementary Information

**Additional file 1.** Quit outcome assessment designed by the study team, administered at day 6, week 6, and 6 months post-target quit date.

**Additional file 2.** Daily diaries developed by the study team, administered daily via REDCap.

**Additional file 3.** Original ethical approval of the trial from CAMH Research Ethics Board.

**Additional file 4.** Official funding document from Canadian Cancer Society.

## Data Availability

The datasets generated or analyzed during the current study are available from the corresponding author upon reasonable request.

## Abbreviations

AE: Adverse event
BHDB-RR: BrainHealth Databank Research Resource
CAMH: Centre for Addiction and Mental Health
HSI: Heaviness of smoking index
IUD: Intrauterine device
MNWS: Minnesota Nicotine Withdrawal Scale
NDS: Nicotine Dependence Services
NRT: Nicotine replacement therapy
PANAS-SF: Positive Affect and Negative Affect Schedule – Short Form
PHI: Personal health identifiers
PHQ-9: Patient Health Questionnaire-9
PI: Principal investigator
PSS-4: Perceived Stress Scale 4
REB: Research Ethics Board
REDCap: Research Electronic Data Capture
SAE: Serious adverse event
SOPs: Standards of operation
STOP: Smoking Treatment for Ontario Patients
