# Supplementary material for: Coordinating smoking cessation treatment with menstrual cycle phase to improve quit outcomes (MC-NRT): study protocol for a randomized controlled trial
Source: Trials. 2023 Apr 1;24:251. doi: 10.1186/s13063-023-07196-1 (PMC10066995; doi:10.1186/s13063-023-07196-1)
Supplement: Supplementary file 1 — Additional file 1. Quit outcome assessment designed by the study team, administered at day 6, week 6, and 6 months post-target quit date. [file 13063_2023_7196_MOESM1_ESM.docx]

**MENSTRUAL CYCLE PHASE NRT TQD STUDY**

APPENDIX K — QUIT OUTCOME

**This questionnaire will ask you about your current quit status. Please answer this questionnaire to the best of your ability and honestly. This questionnaire should take approximately 2 minutes of your time to complete.**

1. **Have you smoked any tobacco cigarettes, even a puff, in the last 7 days?**

Yes No

1. [If YES to 1] **How many tobacco cigarettes do you currently smoke per day?**

[open-number field]

1. [If NO to 1] **Have you smoked any tobacco cigarettes, even a puff, in the last 30 days?** [Note: Only ask for 6-week follow-up]

Yes No
