# Supplementary material for: Coordinating smoking cessation treatment with menstrual cycle phase to improve quit outcomes (MC-NRT): study protocol for a randomized controlled trial
Source: Trials. 2023 Apr 1;24:251. doi: 10.1186/s13063-023-07196-1 (PMC10066995; doi:10.1186/s13063-023-07196-1)
Supplement: Supplementary file 2 — Additional file 2. Daily diaries developed by the study team, administered daily via REDCap. [file 13063_2023_7196_MOESM2_ESM.docx]

**MENSTRUAL CYCLE PHASE NRT TQD STUDY**

APPENDIX F — DAILY DIARY

Please complete at the end of each day starting on your quit date.

1. **Completion date:** [automatically filled field; hidden from participant]
2. **Date of diary entry:** [field code for calendar]
3. **Are you currently on your period?** ⃝ Yes ⃝ No
4. **Total number of cigarettes smoked today:** (if you did not smoke today, please enter 0) [number entry field]
5. [If total number of cigarettes smoked > 0] **How soon after you woke up did you have your first cigarette?**

⃝ Within 5 minutes

⃝ 6 to 30 minutes

⃝ 31 to 60 minutes

⃝ After 60 minutes

1. **Did you use NRT today?** ⃝ Yes ⃝ No

**6a.** [If YES] **Which forms of NRT did you use? Please check all that apply.**

⃝ Patch

⃝ Gum/Lozenge

1. **Did you experience any of the following today?** Please check all that apply.

⃝ Skin Irritation

⃝ Dizziness

⃝ Racing Heartbeat

⃝ Sleep Problems or Unusual Dreams

⃝ Headache

⃝ Nausea

⃝ Mouth or Throat Irritation

⃝ Hiccups

⃝ Jaw Discomfort

⃝ Coughing

⃝ Upset Stomach

⃝ Tender Breasts

⃝ Muscle Aches

⃝ Abdominal Cramps

⃝ Lower Back Pain

⃝ Low Energy, Fatigue

⃝ Fluid Retention

⃝ Joint Pain

⃝ Ache

⃝ Diarrhea or Constipation

⃝ Other: [open-text field]

⃝ Did not experience any of the side effects
