# Supplementary material for: Coordinating smoking cessation treatment with menstrual cycle phase to improve quit outcomes (MC-NRT): study protocol for a randomized controlled trial
Source: Trials. 2023 Apr 1;24:251. doi: 10.1186/s13063-023-07196-1 (PMC10066995; doi:10.1186/s13063-023-07196-1)
Supplement: Supplementary file 3 — Additional file 3. Original ethical approval of the trial from CAMH Research Ethics Board. [file 13063_2023_7196_MOESM3_ESM.pdf]

camh

June 9, 2022

Study Title: Coordinating Smoking Cessation Treatment with Menstrual Cycle Phase to Improve Quit Outcomes: A Randomized Controlled Trial

Principal Investigator: Dr. Laurie Zawertailo, Centre for Addiction and Mental Health

REB #: 022/2022
Review Type: Delegated

Initial Date: June 9, 2022
Expiry Date: June 9, 2023

Study Approval Letter

The Centre for Addiction and Mental Health Research Ethics Board has reviewed this study and has granted approval until the expiry date noted above.

Documents Approved:

- Protocol (Version 1.1 dated 06-May-2022)
- Informed Consent Form (Version dated 06-May-2022)
- Appendix A Behavioural Support Program Storyboard (Version 1.2 dated June 9 2022)
- Appendix B Screening Questionnaire (Version 1.1 dated May 6 2022)
- Appendix C Assessment Questionnaire (Version 1.1 dated May 6 2022)
- Appendix D Emails (Version 1.1 dated May 6 2022)
- DBS Instructions (Version 1.2 dated June 6 2022)
- Appendix F Daily Diary (Version 1.1 dated May 6 2022)
- Appendix G PHQ-9 (Version 1.1 dated May 6 2022)
- Appendix H PANAS (Version 1 dated April 12 2022)
- Appendix I MNWS (Version 1 dated April 12 2022)
- Appendix J My Change Plan Mobile Application (Version 1.1 dated March 21 2022)
- Appendix K Quit Outcome (Version 1.1 dated May 6 2022)
- Appendix L Satisfaction Survey (Version 1.1 dated May 9 2022)
- Appendix M 6m Follow-Up Questionnaire (Version 1.2 dated June 6 2022)
- Appendix N Contact Information (Version 1 dated April 1 2022)
- Appendix O NRT Info Sheet (Version 1.1 dated March 21 2022)
- Appendix P Target Quit Date (Version 1.1 dated May 6 2022)

- Appendix Q Video Scripts (Version 1 dated April 14 2022)
- Appendix R PSS4 (Version 1 dated April 14, 2022)
- Appendix S Handout (Version 1 dated April 14 2022)
- Appendix T Consent FAQs (Version 1 dated May 6 2022)
- Appendix U Website mockup (Version 1.0 dated April 11 2022)
- Advertisement 1 (Version 1.1 dated 21MAR2022)
- Advertisement 2 (Version 1.1 dated 21MAR2022)
- Advertisement 3 (Version 1.1 dated 21MAR2022)
- Advertisement 4 (Version 1.1 dated 21MAR2022)
- Advertisement 5 (Version 1.1 dated 21MAR2022)
- Advertisement 6 (Version 1.1 dated 21MAR2022)
- Advertisement 7 (Version 1.1 dated 21MAR2022)
- Advertisement 8 (Version 1.1 dated 21MAR2022)
- Ad Text (Version 1.1 dated March 21 2022)

Documents Acknowledged:

- Budget

Please note the following:

- This study must be conducted as outlined in the REB approved materials, and in accordance with CAMH policies and procedures, the TCPS2 (2nd edition of the Tri-Council Policy Statement: Ethical Conduct for Research Involving Humans), the provisions of the Ontario Personal Health Information Protection Act and its applicable Regulations, and with all other applicable laws, regulations or guidelines
- No deviations from, or changes to, the protocol should be initiated without prior written approval from the CAMH REB, except when necessary to eliminate immediate hazard(s) to study participants
- Ethics approval must be renewed prior to the expiry noted above - failure to do so will result in an immediate suspension of ethics approval

REB members with a conflict of interest on a study do not participate in the discussion, deliberation or decision on such studies.

The Centre For Addiction and Mental Health Research Ethics Board (CAMH REB) operates in compliance with, and is constituted in accordance with, the requirements of the Tri-Council Policy Statement: Ethical Conduct for Research Involving Humans (TCPS 2), the International Conference on Harmonisation Good Clinical Practice Consolidated Guideline (ICH GCP), Part C, Division 5 of the Food and Drug Regulations, Part 4 of the Natural Health Products Regulations, Part 3 of the Medical Devices Regulations, and the provisions of the Ontario Personal Health Information Protection Act (PHIPA 2004) and its applicable

regulations. The CAMH REB is qualified through the CTO REB Qualification Program and is registered with the U.S. Department of Health and Human Services (DHHS) Office for Human Research Protection (OHRP).

Yours sincerely,

Dr. Robert Levitan
Chair
Research Ethics Board
Centre for Addiction and Mental Health
E-mail: Robert.levitan@camh.ca
Telephone: 416-535-8501 x 34020
