# Supplementary material for: Coordinating smoking cessation treatment with menstrual cycle phase to improve quit outcomes (MC-NRT): study protocol for a randomized controlled trial
Source: Trials. 2023 Apr 1;24:251. doi: 10.1186/s13063-023-07196-1 (PMC10066995; doi:10.1186/s13063-023-07196-1)
Supplement: Supplementary file 4 — Additional file 4. Official funding document from Canadian Cancer Society. [file 13063_2023_7196_MOESM4_ESM.pdf]

Canadian Cancer Society
55 St. Clair Avenue West, Suite 300
Toronto, ON M4V 2Y7

NOTIFICATION OF AWARD

Grant #: 707321-1
Grant Class: Initial Application

Grant Type: Challenge Grants - 2022
Funder: Canadian Cancer Society

**Principal Investigator:** Laurie Zawertailo	**Institution:** Centre for Addiction and Mental Health
**Co-Applicant:** Rosa Dragonetti	**Institution:** Centre for Addiction and Mental Health
**Co-Applicant:** Elad Mei-Dan	**Institution:** North York General Hospital
**Co-Applicant:** Osnat Melamed	**Institution:** Centre for Addiction and Mental Health
**Co-Applicant:** Nadia Minian	**Institution:** Centre for Addiction and Mental Health
**Co-Applicant:** Peter Selby	**Institution:** Centre for Addiction and Mental Health
**Co-Applicant:** Scott Veldhuizen	**Institution:** Centre for Addiction and Mental Health
**Additional Author:** Sophia Attwells	**Institution:** Centre for Addiction and Mental Health
**Additional Author:** Elise Tanzini	**Institution:** Centre for Addiction and Mental Health
**Additional Author:** Sabrina Voci	**Institution:** Centre for Addiction and Mental Health

Research Project Title: Coordinating smoking cessation treatment with menstrual cycle phase to improve quit outcomes: a randomized controlled trial

Payments will be made to: Centre for Addiction and Mental Health

This is a 3 year grant with a start date of 01/01/2022.
Payments will be made in quarterly installments beginning on the start date of the grant and on each subsequent anniversary date until the final installment is made. Payments are subject to satisfaction of any encumbrances holding payment, as well as receipt of annual scientific reports, as applicable.See below for additional payment information.
Annual scientific reports are required 15 days after the grant year end. Post grant reports are required two years after the end date of the grant. Annual Statements of Account are required 60 days after the grant year end.

Grant Awarded	2021/2022	2022/2023	2023/2024
Supplies and Expenses	120,000	120,000	120,000
Salaries and Wages	30,000	30,000	30,000
Total	150,000	150,000	150,000

CRITICAL FUNDING INFORMATION

Any encumbrances associated with this award are outlined below.

The funds for this grant have been provided by the Canadian Cancer Society through donations from the public. Any publications or communications that result from this project should include the following acknowledgement: "This research is funded by the Canadian Cancer Society (grant #xxxxxx)."

This Notification of Award (NOA) covers the ENTIRE term of the award. Please keep it in a safe place for future reference. Further NOA's will only be produced if there is a change in the amount or condition of the award. A new NOA will be forwarded to you prior to any change taking effect.

This grant must be accepted in the CCS online system (EGrAMS).

Acceptance of this award indicates acknowledgement of your understanding of the terms and conditions as described on our website (https://cancer.ca/en/research/for-researchers/managing-your-grant) and under the specific Program Description for this competition. Contravention of CCS policies may result in serious implications including the termination of this grant.

NO FUNDS will be released until the approval from the local biohazard committee for the project named above is provided. Certificates that expire before the start date of this grant are not acceptable.

NO FUNDS will be released until the approval by an appropriate committee that the study named above has been found acceptable from an ethical point of view is provided. Certificates that expire before the start date of this grant are not acceptable.

Funds for this project will not be released until appropriate evidence that the PI has registered/enrolled for bio-specimen collection with a quality assurance program is submitted to CCS. Please review the CCS Research website (http://www.cancer.ca/en/research/policies-and-administration/policies/human-samples-policy/) for more information.

All grants and awards are at all times conditional upon and subject to the availability of funds to the Canadian Cancer Society. In addition, the Canadian Cancer Society reserves the right to terminate, at any time, and without cause, any award or grant.

All awards are also subject to the conditions set forth on our website.

per ____________________________________

Canadian Cancer Society

AMENDMENT HISTORY

Date	Amendment Type	Amendment Detail
02/04/2022	Participants	The lsit of participants in this grant have changed
